# The Association Between Obesity, Obstructive Sleep Apnea, and Postoperative Complications in Breast Reduction Patients: A Propensity Score-Matched Analysis

**DOI:** 10.1093/asjof/ojag026

**Published:** 2026-02-10

**Authors:** Nir Zontag, Ron Skorochod, Yoram Wolf

## Abstract

**Background:**

Obstructive sleep apnea (OSA) is associated with increased risk for several comorbidities, with the most prominent being obesity. Obesity is strongly linked to the development of macromastia, a primary indication for breast reduction. It is highly important to assess whether OSA serves as an independent risk factor for postoperative complications.

**Objectives:**

The aim of this study was to assess the rates of short- and long-term complications following breast reduction in obese patients (BMI >30) with and without documented OSA.

**Methods:**

A retrospective cohort analysis was conducted using the TriNetX Global Collaborative Network. Patients >18 years, with BMI >30, who underwent breast reduction were divided into 2 groups: those with documented OSA before surgery and those with no history of OSA. Propensity score matching (PSM) was applied to balance demographic and clinical variables. Primary outcomes included short-term postoperative complications at 30, 60, and 90 days. Secondary outcomes included long-term complications after 1 and 2 years.

**Results:**

After 1:1 PSM, each cohort consisted of 3414 patients. Within 30 days postsurgery, patients in the OSA cohort had a significantly increased risk of surgical-site infection (risk ratio [RR]: 1.444, *P* = .02), readmission (RR: 1.512, *P* = .03), inpatient hospitalization (RR: 1.326, *P* = .04), and opioid use (RR: 1.122, *P* < .0001) compared with the control group. Results remained consistent at 60 and 90 days postsurgery. For long-term outcomes, OSA patients had reduced rates of breast deformity at 1 year and surgical revision rates at 2 years.

**Conclusions:**

OSA is associated with increased risk for short-term postoperative complications following breast reduction surgery. However, OSA was paradoxically associated with lower rates of breast deformity and surgical revision.

**Level of Evidence: 3 (Therapeutic):**

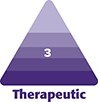

Obstructive sleep apnea (OSA) is a sleep disorder characterized by intermittent obstruction of the upper respiratory tract. This leads to periodic cessations or decreases in airflow during breathing efforts, which in turn poses a risk for the development and progression of several comorbidities, such as hypertension, myocardial infarction, heart failure, arrhythmias, stroke, and metabolic syndrome.^1^

Obesity is a common comorbidity worldwide, as estimations suggest >2 billion people are affected.^2^ Macromastia, a term that signifies an abnormally enlarged breast, has been linked to obesity directly. Macromastia has a great impact on the quality of life, both physically and psychologically, by causing back pain, degenerative spinal changes, headaches, low self-esteem, negative body image, and associations with depression and anxiety.^3-6^ It can be managed nonsurgically, through weight loss or using special brassieres, or surgically through breast reduction.^6^ Breast reduction surgery is the primary definitive treatment indicated for macromastia and has shown great improvement in many aspects of quality of life, including pulmonary function, which can be impaired because of macromastia, highlighting the beneficial impact of the procedure.^7,8^

Recently, studies have linked OSA to higher postoperative complications across several disciplines.^9-12^ Because overweight is a known risk factor for macromastia, and given the high burden of OSA among overweight individuals, we find it crucial to further investigate the effect of OSA on breast reduction surgery.^13,14^ This study aims to assess whether OSA poses a risk for postoperative short- and long-term complications after breast reduction, using a large global electronic medical record (EMR) network.

## METHODS

We performed a retrospective cohort study using the TriNetX (TriNetX LLC, Cambridge, MA) Global Collaborative Network, which, as of October 2025, includes de-identified EMRs from 163 healthcare organizations. Data extraction relied on Current Procedural Terminology, Anatomical Therapeutical Chemical, and International Classification of Diseases, Revision 10 (ICD-10) codes.

The analysis process involves 2 main steps: (1) defining the cohorts based on specific query criteria and (2) setting up and executing the analysis. Setting up the analysis includes defining the index event, outcome criteria, and time frame. Outcome comparisons were performed using risk ratio (RR) analysis. Additionally, cohort characteristics balanced through propensity score matching (PSM) are presented in the Statistical Analysis section.

### Ethical Statement

All data within the TriNetX platform are de-identified in compliance with the Health Insurance Portability and Accountability Act (HIPAA) Privacy Rule. Because this study involved the analysis of retrospective, de-identified data, it is exempt from IRB oversight and the requirement for informed consent under HIPAA regulations. The data reviewed is a secondary analysis of existing data, does not involve intervention or interaction with human participants, and is de-identified per the de-identification standard defined in Section §164.514(a) of the HIPAA Privacy Rule. The process by which the data are de-identified is attested to through a formal determination by a qualified expert as defined in Section §164.514(b)(1) of the HIPAA Privacy Rule.

### Cohorts

**Figure 1.**
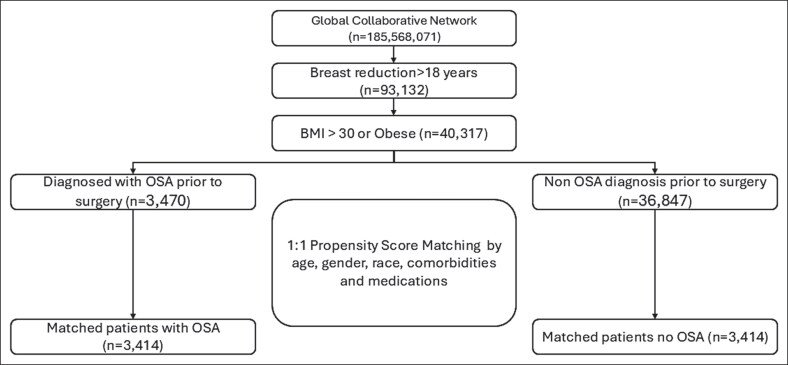
Flowchart illustrating the cohort selection process from the TriNetX global electronic health record network and the 1:1 propensity score matching procedure.

We defined 2 cohorts of patients aged 18 and older, with a BMI >30. The exposure cohort included patients who underwent breast reduction with documented OSA before surgery, and the control cohort consisted of patients with no history of OSA, who underwent the same surgery (Figure 1). Supplemental Table 1 summarizes the complete list of codes for inclusion and exclusion criteria. The index event was defined as the date of surgery.

### Outcomes

Our primary aim was to assess short-term complications following breast surgery. To do so, we used 3 postoperative time intervals: at 30, 60, and 90 days after surgery. For short-term outcomes, we included surgical-related complications, such as surgical-site infection (SSI), wound dehiscence, hematoma, seroma, readmission, inpatient hospitalization, opioid use, and any surgical-site complications. Because OSA has been associated with systemic conditions, we have also evaluated acute kidney failure, pulmonary embolism (PE), deep vein thrombosis (DVT), and pneumonia.^1,15^ Our second aim was to assess long-term complications by assessing the risk of deformity of the reduced breast and surgical revision at 2 time points: 1 and 2 years postsurgery.

### Statistical Analysis

PSM was performed using a 1:1 ratio using logistic regression with a caliper of 0.01. The cohorts were balanced on variables measured from 1 year to 1 day before the index event, including demographics (age, sex, race, and ethnicity), comorbidities (hypertensive diseases, chronic ischemic heart disease, Types 1 and 2 diabetes mellitus, BMI, obstructive pulmonary diseases, cerebrovascular diseases, chronic kidney disease, and liver disease), nicotine dependence, and alcohol-related disorders. Variables were considered significantly different between groups if the standard difference was ≥0.1 and the *P*-value was < .05. The complete list of variables used for PSM is provided in Supplemental Table 2. We performed all the statistical analyses using the TriNetX platform. For all outcomes, risks, RRs, and 95% CIs were assessed. A *P*-value <.05 was considered statistically significant.

## RESULTS

A total of 40,317 patients met the inclusion criteria. OSA cohort included patients with documented OSA before breast reduction surgery, whereas the control cohort included patients with no history or documentation of OSA who underwent the same surgery. After matching, each cohort included 3414 patients. Table 1 summarizes the baseline characteristics before and after matching.

**Table 1.** Baseline Characteristics of Patients With OSA Included in the Breast Reduction Cohort, With Comparison to the Corresponding Control Group

Characteristics, *n* (%)	Before matching		*P*-value	After matching		*P*-value
	OSA cohort (*n* = 3470)	Control cohort (*n* = 36,847)		OSA cohort (*n* = 3414)	Control cohort (*n* = 3414)	
Age at index, years (mean ± SD)	51.3 ± 12.9	42.3 ± 15.0	<.001	51.2 ± 12.9	52.2 ± 13.4	<.001
Sex						
Female	3428 (98.8%)	35,991 (98.7%)	.51	3374 (98.8%)	3383 (99.1%)	.28
Male	39 (1.1%)	400 (1.1%)	.88	37 (1.1%)	29 (0.8%)	.32
Race						
White	2115 (61.0%)	20,541 (56.3%)	<.001	2072 (60.7%)	2143 (62.8%)	.08
Black or African American	977 (28.2%)	11,073 (30.4%)	.01	967 (28.3%)	910 (26.7%)	.12
Asian	31 (0.9%)	317 (0.9%)	.88	31 (0.9%)	20 (0.6%)	.12
Ethnicity						
Not Hispanic or Latino	2663 (76.7%)	28,095 (77.0%)	.72	2620 (76.7%)	2621 (76.8%)	.98
Hispanic or Latino	266 (7.7%)	3384 (9.3%)	<.001	263 (7.7%)	249 (7.3%)	.52
Comorbidities						
Hypertensive diseases	1968 (56.7%)	7779 (21.3%)	<.001	1912 (56.0%)	1940 (56.8%)	.49
Type 2 diabetes mellitus	917 (26.4%)	2614 (7.2%)	<.001	870 (25.5%)	834 (24.4%)	.31
Type 1 diabetes mellitus	56 (1.6%)	159 (0.4%)	<.001	54 (1.6%)	45 (1.3%)	.36
Chronic ischemic heart disease	289 (8.3%)	675 (1.9%)	<.001	271 (7.9%)	256 (7.5%)	.50
Cerebrovascular diseases	175 (5.0%)	482 (1.3%)	<.001	164 (4.8%)	157 (4.6%)	.69
Chronic kidney disease	227 (6.5%)	509 (1.4%)	<.001	207 (6.1%)	177 (5.2%)	.12
Other chronic obstructive pulmonary disease	228 (6.6%)	367 (1.0%)	<.001	198 (5.8%)	151 (4.4%)	.01
Other diseases of the liver	470 (13.5%)	1362 (3.7%)	<.001	442 (12.9%)	412 (12.1%)	.27
Substance use						
Alcohol-related disorders	71 (2.0%)	356 (1.0%)	<.001	66 (1.9%)	63 (1.8%)	.79
Nicotine dependence	314 (9.0%)	1757 (4.8%)	<.001	304 (8.9%)	281 (8.2%)	.32
BMI						
BMI, mean (SD)	36.7 (6.7)	34.5 (5.2)	<.001	36.6 (6.7)	36.3 (6.1)	.02
BMI 30-40 kg/m^2^	2825 (81.4%)	31,288 (85.8%)	<.001	2780 (81.4%)	2806 (82.2%)	.41
BMI 40-45 kg/m^2^	1392 (40.1%)	6205 (17.0%)	<.001	1339 (39.2%)	1351 (39.6%)	.77
BMI 45-50 kg/m^2^	770 (22.2%)	2205 (6.0%)	<.001	724 (21.2%)	698 (20.4%)	.44

SD, standard deviation.

At 30 days postoperation, OSA patients that undergo breast reduction surgery had an increased risk for SSI (RR: 1.736, *P* = .02), hematoma (RR: 1.466, *P* = .02), readmission (RR: 1.512, *P* = .03), inpatient hospitalization (RR: 1.326, *P* = .04), opioid use (RR: 1.122, *P* < .0001), and overall surgical-site complication rates (RR: 1.338, *P* = .001) in comparison to control patients. However, there was no significant difference in the rates of wound dehiscence, seroma, AKI, PE, DVT, or pneumonia (Table 2).

**Table 2.** Postoperative Complications of Patients With OSA Compared With Patients Without OSA at Postoperative Day 30

	OSA cohort (*n* = 3414)	Control cohort (*n* = 3414)	Risk ratio	95% CI	*P*-value
Surgical-site infection	91	63	1.444	1.051-1.985	.02
Wound dehiscence	99	77	1.286	0.958-1.725	.09
Hematoma	85	58	1.466	1.053-2.039	.02
Seroma	33	20	1.650	0.949-2.870	.07
Readmission	65	43	1.512	1.031-2.216	.03
Inpatient hospitalization	122	92	1.326	1.016-1.731	.04
Opioid use	1442	1285	1.122	1.059-1.190	<.0001
Any surgical-site complications	261	195	1.338	1.119-1.601	.001
Acute kidney failure	22	15	1.467	0.762-2.822	.25
PE	24	19	1.263	0.693-2.302	.44
DVT	26	20	1.3	0.727-2.324	.38
Pneumonia	18	16	1.125	0.575-2.202	.73

DVT, deep vein thrombosis; OSA, obstructive sleep apnea; PE, pulmonary embolism.

After 60 days of surgery, the OSA cohort continued to show increased complication rates with SSI (RR: 1.455, *P* = .01), wound dehiscence (RR: 1.342, *P* = .02), readmission (RR: 1.508, *P* = .01), inpatients hospitalization (RR: 1.305, *P* = .03), opioid use (RR: 1.085, *P* = .004), and in the overall surgical-site complication rates (RR: 1.282, *P* = .001) in comparison to patients with no history of OSA. Table 3 summarizes the complete measured outcomes.

**Table 3.** Postoperative Complications of Patients With OSA Compared With Patients Without OSA at Postoperative Day 60

	OSA cohort (*n* = 3414)	Control cohort (*n* = 3414)	Risk ratio	95% CI	*P*-value
Surgical-site infection	128	88	1.455	1.113-1.900	.01
Wound dehiscence	153	114	1.342	1.058-1.702	.02
Hematoma	95	78	1.218	0.906-1.637	.19
Seroma	43	32	1.344	0.852-2.118	.20
Readmission	92	61	1.508	1.095-2.077	.01
Inpatient hospitalization	154	118	1.305	1.032-1.651	.03
Opioid use	1515	1396	1.085	1.027-1.147	.004
Any surgical-site complications	359	280	1.282	1.105-1.488	.001
Acute kidney failure	31	27	1.148	0.687-1.919	.60
PE	32	22	1.455	0.847-2.498	.17
DVT	31	30	1.033	0.627-1.703	.90
Pneumonia	29	23	1.261	0.731-2.175	.40

DVT, deep vein thrombosis; OSA, obstructive sleep apnea; PE, pulmonary embolism.

At 90 days postsurgery, these increased rates persisted, with patients in the OSA group having a higher risk of SSI (RR: 1.432, *P* = .01), wound dehiscence (RR: 1.278, *P* = .04), readmission (RR: 1.486, *P* = .01), inpatient hospitalization (RR: 1.281, *P* = .03), opioid use (RR: 1.082, *P* = .004), and overall surgical-site complication rates (RR: 1.259, *P* = .001) in comparison to matched control patients. The remaining outcomes measured were not significantly different, as detailed in Table 4.

**Table 4.** Postoperative Complications of Patients With OSA Compared With Patients Without OSA at Postoperative Day 90

	OSA cohort (*n* = 3414)	Control cohort (*n* = 3414)	Risk ratio	95% CI	*P*-value
Surgical-site infection	136	95	1.432	1.106-1.852	.01
Wound dehiscence	161	126	1.278	1.017-1.605	.04
Hematoma	102	85	1.200	0.903-1.594	.21
Seroma	48	38	1.263	0.828-1.928	.28
Readmission	110	74	1.486	1.111-1.988	.01
Inpatient hospitalization	173	135	1.281	1.028-1.597	.03
Opioid use	1566	1447	1.082	1.026-1.142	.004
Any surgical-site complications	389	309	1.259	1.093-1.450	.001
Acute kidney failure	37	32	1.156	0.722-1.851	.55
PE	35	26	1.346	0.812-2.231	.25
DVT	34	38	0.894	0.565-1.418	.64
Pneumonia	33	27	1.222	0.737-2.028	.44

DVT, deep vein thrombosis; OSA, obstructive sleep apnea; PE, pulmonary embolism.

For long-term outcomes, we assessed after 1- and 2-year rates of breast deformity and surgical revisions. After 1-year OSA group had a significantly decreased risk for deformity (RR: 0.725, *P* = .03), whereas surgical revision rates were not different. Two years after surgery, deformity was not significantly different, whereas surgical revision rate was significantly decreased in patients with OSA (RR: 0.75, *P* = .04; Tables 5, 6).

**Table 5.** Postoperative Complications of Patients With OSA Compared With Patients Without OSA 1 Year After Surgery

	OSA cohort (*n* = 3414)	Control cohort (*n* = 3414)	Risk ratio	95% CI	*P*-value
Deformity of reduced breast	74	102	0.725	0.54-0.975	.03
Surgical revision of reduced breast	62	80	0.775	0.558-1.076	.13

OSA, obstructive sleep apnea.

**Table 6.** Postoperative Complications of Patients With OSA Compared With Patients Without OSA 2 Years After Surgery

	OSA cohort (*n* = 3414)	Control cohort (*n* = 3414)	Risk ratio	95% CI	*P*-value
Deformity of reduced breast	97	117	0.829	0.636-1.081	.17
Surgical revision of reduced breast	84	112	0.75	0.567-0.991	.04

OSA, obstructive sleep apnea.

## DISCUSSION

The perioperative risk associated with OSA has been well described in current literature. OSA patients suffer from difficult airway management, increased susceptibility to opioid-induced respiratory depression, and altered ventilation and oxygenation mechanisms.^16-21^

As a direct derivative, numerous studies reported an increase in the risk for medical complications in OSA patients undergoing various surgical procedures, namely DVT, PE, cardiovascular disease, and pulmonary-related complications.^9,11,22-24^

However, the possible association between OSA and surgical complications has yet to be defined. Previous studies suggested that in joint arthroplasty, OSA is associated with increased risk for short-term surgical complications, hospital readmissions, prolonged length of stay, and intensive care unit admission.^10,12,25^ Other studies claimed the opposite and found no association between OSA and an increase in the rate of adverse events.^12,26,27^

In this multi-institutional propensity-matched cohort study, OSA was found to be statistically associated with an increased risk of adverse outcomes following breast reduction surgery in obese patients. Patients with documented OSA experienced significantly higher rates of SSI, hematoma, readmission, inpatient hospitalization, and opioid use in the short-term postoperative period, compared with matched non-OSA patients. Interestingly, long-term follow-up demonstrated a relative reduction in breast deformity and frequency of revision procedures at 1 and 2 years postoperatively.

Findings from our study delineate a complex relationship between OSA and breast reduction outcomes; although the immediate postoperative period adverse events are more frequent, the ultimate reconstructive or aesthetic outcomes remain comparable or superior.

The presumed effect of OSA on adverse events is most likely multifactorial. Repeated nocturnal hypoxemia and hypercapnia trigger systemic inflammation, oxidative stress, and endothelial dysfunction, all of which impair microcirculation and delay wound healing. Additionally, chronic sympathetic activation, characteristic of OSA, contributes to hypertension, insulin resistance, and immune dysregulation.^28-31^ Although the cohorts were matched for hypertension and insulin resistance, patients with OSA have been linked to treatment-resistant hypertension and more difficult to control insulin levels.^32-34^ The aggregative effect plays a pivotal role in the increased susceptibility to adverse events noted in our study.

Moreover, our findings suggest no significant differences in the incidence of PE, DVT, or pneumonia within 90 days following surgery, which may indicate a potential short-term protective effect of breast reduction. Studies have shown OSA is associated with increased risk of pulmonary and thromboembolic complications, likely through impaired lung function and nocturnal sympathetic and hemodynamic changes.^35,36^ This is particularly important because breast reduction was found to improve the restrictive pulmonary impairment caused by macromastia, highlighting whether it also improves OSA and its respiratory complications.^8^ We do acknowledge that our study could not directly measure respiratory function and OSA severity; the observed outcomes imply a potential improvement in respiratory-related complications.

Despite the short-term disadvantages, long-term outcomes in OSA patients were interestingly superior. At 1-year, postoperatively, deformity of the reduced breast was significantly less common among patients, and by 2 years, the rate of surgical revision was also reduced. The evident conundrum, in our hypotheses, is likely attributed to procedural, psychological, and study-design-related factors. Surgeons aware of OSA implications may adopt more conservative surgical approaches and subject the patients to more rigorous or frequent follow-up appointments, leading to early detection and resolution of complications.

Furthermore, individuals with OSA, particularly those under chronic medical care, may interact more regularly with healthcare professionals, which could lead to better adherence to postoperative follow-up visits and may improve compliance with postoperative restrictions and wound-care instructions. And last, the retrospective nature of the cohort could lead to selection bias, and the reduced revision rate could represent surgeons being less inclined to predispose these patients to additional anesthesia and revision surgeries.

From a clinical standpoint, findings from our study carry critical implications for both the preoperative consultation of patients and the intraoperative management. Given the high prevalence of OSA among patents with obesity, and the known volume of undiagnosed cases, routine preoperative screening using validated tools should be considered in all patients undergoing breast reduction surgery. Furthermore, education of patients with OSA about the presumed relative risk increase is critical for ensuring informed consent and patient satisfaction. Open discussion should be conducted with OSA patients regarding postoperative complications risk their condition poses alongside the upsides of the surgery.

Identification of OSA patients can lead surgeons to change surgical planning, implement different techniques, and dictate the optimal postoperative care to minimize serious adverse events.

The main strengths of our study include its large sample size, multicenter design, rigorous statistical pairwise matching, and long-term data on patients’ rehabilitation course. The longitudinal follow-up enabled using the TriNetX database provides a real-world representation of breast reduction practice across multiple institutions and states. Conversely, utilization of the TriNetX database serves as the main limitation of our study. Because the database is limited to institutions belonging to the network, patients seeking medical attention in other facilities may not be included in statistical analyses, which can lead to misclassification bias. Additionally, use of coded and anonymized patient data and not open medical files limits access to information outside the query and can result in coding errors impacting data quality. Moreover, because we have solely relied on ICD codes, this might have caused potential biases related to data accuracy, consistency, and variability in coding practices across institutions. The small number of men, who were included in our analysis, likely reflecting variation in sex coding across centers, such as including transmen. Furthermore, we were unable to further stratify the OSA cohort by severity. Detailed clinical measures such as apnea-hypopnea index scores, which are necessary to classify OSA severity, were not available in the dataset and therefore could not be incorporated into the final analysis. In addition, several confounding factors could also not be addressed using the coding system, including resection weight and the type of anesthesia. Similarly, indication of revision surgery and magnitude of breast deformity were not reported and were solely represented as binary outcomes. Also, we acknowledge that for long-term outcomes, major revisions and deformities were primarily captured, because minor revisions were mainly performed in office settings and may not have been consistently recorded.

Future research should aim to investigate the dose–response relationship between OSA severity and adherence to therapy and surgical risk. A better understanding of the association can serve as a bridge to improved patient preparation for surgery and optimal patient care. Integration of OSA screening questionnaires into touring preoperative consultations can examine the magnitude of underdiagnosed OSA in breast reduction patients and thus improve their outcomes and care.

## CONCLUSIONS

In conclusion, results from the study indicate that OSA is a significant risk factor for early postoperative complications following breast reduction surgery in obese patients. On the contrary, revision rates and long-term breast deformity are equivocal or lower in OSA patients. These findings serve as the first body of evidence regarding the impact of OSA on breast reduction adverse events and highlight the importance of perioperative vigilance and multidisciplinary care of OSA among breast reduction candidates.

## Supplementary Material

ojag026_Supplementary_Data
