# Supplementary material for: The Association Between Obesity, Obstructive Sleep Apnea, and Postoperative Complications in Breast Reduction Patients: A Propensity Score-Matched Analysis
Source: Aesthet Surg J Open Forum. 2026 Feb 10;8:ojag026. doi: 10.1093/asjof/ojag026 (PMC12968777; doi:10.1093/asjof/ojag026)
Supplement: ojag026_Supplementary_Data [file ojag026_supplementary_data.docx]

**Supplemental Table 1**. Current Procedural Terminology (CPT), Anatomical Therapeutical Chemical (ACT), and International Classification of Diseases, Revision 10 (ICD-10) Codes That Were Used For Patient Selection

| Surgical Procedure | CPT Code |
| --- | --- |
| Breast reduction | CPT:19318 |
| Exposure cohort |  |
| Must have:  BMI: TNX:9083 (at least 30.00 kg/m2).  AND  Obstructive sleep apnea (adult) (pediatric): UMLS:ICD10CM:G47.33) | |
| Control cohort |  |
| Must have:  BMI: TNX:9083 (at least 30.00 kg/m2).  Cannot have:  Obstructive sleep apnea (adult) (pediatric): UMLS:ICD10CM:G47.33) | |
| Outcomes and Complications | ICD-10 and CPT Codes |
| Surgical site infection | ICD10CM: T81.4, T81.4XXA |
| Wound dehiscence | ICD10CM:T81.3 |
| Hematoma | ICD10CM: M96.84, M96.83, L76.3, L76.32, M79.81 |
| Seroma | ICD10CM : L76.34, L76.33, M96.842, M96.843 |
| Readmission | CPT: 99221, 99222, 99223 |
| Inpatient hospitalization | CPT: 1013659 |
| Opioid use | VA:CN101 |
| Any surgical site complications | ICD10CM: T80-T88 |
| Acute Kidney Injury | N17 |
| Deep Vein Thrombosis | I82.4, I82.6 |
| Pulmonary Embolism | I26 |
| Pneumonia | J12-18 |
| Deformity of reduced breast | ICD10CM:N65.0 |
| Revision of reduced breast | CPT:19380 |

**Supplemental Table 2**. Variables of the Propensity Score Matching

| Code | Description |
| --- | --- |
| I10-I15 | Hypertensive diseases |
| I25 | Chronic ischemic heart disease |
| E11 | Type 2 diabetes mellitus |
| E10 | Type 1 diabetes mellitus |
| E66 | Overweight and obesity |
| J44 | Other chronic obstructive pulmonary disease |
| I60-I69 | Cerebrovascular diseases |
| K76 | Liver disease, unspecified |
| N18 | Chronic kidney disease (CKD) |
| F10 | Alcohol related disorders |
| F17 | Nicotine dependence |
| 39156-5 | Body Mass Index |
